# Spelling Error

**DOI:** 10.1001/jamanetworkopen.2021.35416

**Published:** 2021-10-19

In the Invited Commentary titled “On the Lack of Native Hawaiian and Pacific Islander Individuals in the Physician Workforce,”^1^ published September 20, 2021, Kekoa Taparra’s name was spelled Tappara throughout. This article has been corrected.^1^

## References

[1] Mori WS. On the lack of Native Hawaiian and Pacific Islander individuals in the physician workforce. JAMA Netw Open. 2021;4(9):e2125399. doi:10.1001/jamanetworkopen.2021.25399 34542622

